# Predictive value of a nomogram model for adverse outcomes in very low birth weight infants with patent ductus arteriosus: A prospective study

**DOI:** 10.3389/fped.2023.1131129

**Published:** 2023-04-11

**Authors:** Xuan Sun, Ling Chen, Jinzhi Gao

**Affiliations:** Department of Pediatrics, Tongji Hospital, Tongji Medical College, Huazhong University of Science and Technology, Wuhan, China

**Keywords:** very low birth weight, patent ductus arteriosus, adverse outcomes, nomogram, echocardiography, NT-ProBNP

## Abstract

**Objective:**

To establish a nomogram model incorporating markers of echocardiography and N-terminal pro brain natriuretic peptide (NT-proBNP) for predicting adverse outcomes of patent ductus arteriosus (PDAao) in very low birth weight infants and to evaluate the predictive values of the model.

**Methods:**

A prospective study was conducted for very low birth weight infants who were admitted from May 2019 to September 2020. An echocardiogram and blood NT-proBNP test were carried out in the first 48 h after birth, and the arterial duct remained open in all patients. Other data collected included clinical symptoms and infant characteristics. A nomogram model was established to predict the risk of PDAao (including severe BPD, IVH, NEC or death). Internal verifications were performed for the nomogram, and the discrimination and calibration of the model were evaluated by the C-index and calibration curve.

**Results:**

Eighty-two infants were enrolled and divided into an adverse outcome (AO) group and normal outcome (NO) group with 41 patients in each group. PDA diameter, PDA maximum flow velocity, left atrium diameter/aortic diameter (LA/AO) ratio and NT-proBNP level were independent risk factors for PDAao and were included in the nomogram model. The model presented good discrimination with a C-index of 0.917 (95% CI 0.859–0.975). The calibration curves in showed high consistency and indicated good **Correspondence:** between the event incidence predicted by the nomogram model and the true incidence of PDAao.

**Conclusion:**

The nomogram model incorporating the PDA diameter, PDA maximum flow velocity, LA/AO ratio and NT-proBNP level in the first 48 h could early predict the later occurrence of PDAao in very low birth weight infants.

## 1. Introduction

Patent ductus arteriosus (PDA) is the most common complication in preterm neonates (1, 2), especially in very low birth weight (VLBW) infants, with an incidence of 33% (3). Persistent left-to-right shunts can lead to an increase in pulmonary blood flow and a decrease in systemic blood flow (4, 5), and hemodynamically significant PDA (hsPDA) is associated with increased mortality and several significant morbidities, including intestinal perforation and necrotizing enterocolitis (NEC), intraventricular hemorrhage (IVH), prolonged respiratory support, bronchopulmonary dysplasia (BPD) and even death (6–11). Therefore, the rapid recognition of severe PDA is important, as its presence is a major cause of morbidity in VLBW infants.

Echocardiography is the gold standard bedside investigation to diagnose PDA, which can be used to estimate shunt volume and assess its hemodynamic significance and can also help in assessing the hemodynamic impact from pulmonary overcirculation and systemic hypoperfusion due to shunt volume (12–14). B-type natriuretic peptide (BNP) is synthesized and secreted by the ventricular myocardium and is used in the diagnosis and management of congestive heart failure (15–17). N-terminal proBNP (NT-proBNP), which is cleaved by proBNP, has a longer half-life than BNP and is therefore preferred for clinical use (18, 19). A few studies have reported on the application of plasma NT-proBNP levels in newborns, mainly its use in the diagnosis of hsPDA and congestive heart failure (20, 21). Studies have shown that echocardiographic parameters such as the PDA diameter, left atrium diameter/aortic diameter (LA/AO) ratio and left ventricular output in combination with the plasma NT-proBNP level may change significantly in the early stage of PDA (12, 22, 23).

The aim of this study was to establish an early prediction model of adverse outcomes of PDA (PDAao) by echocardiographic parameters and NT-proBNP levels and to predict the possibility of PDAao in VLBW infants to provide references for early clinical intervention in PDA.

## 2. Methods

### 2.1. Patients

This prospective study was conducted at our level-three neonatal intensive care unit, Tongji hospital, Huazhong University of Science and Technology, China, from May 2019 to September 2020. Infants with a birth weight less than 1500 g were eligible for inclusion in the study. All candidates underwent an echocardiographic examination and plasma NT-proBNP test during the first 48 h of life, and echocardiography indicated that the arterial duct remained open in all patients. We excluded neonates with chromosomal abnormalities, congenital malformations of the digestive or urinary tract, inherited metabolic disorders, or severe congenital heart malformations (i.e., pulmonary atresia, tetralogy of fallot). Patients for whom treatment was stopped during hospitalization were also excluded.

Patients were allocated into two groups, an adverse outcome group (AO group, patients with adverse outcomes of PDA) and a normal outcome group (NO group, patients without adverse outcomes of PDA). Adverse outcomes of PDA included severe BPD, grade IV with IVH, NEC Bell stage II or above, and death.

### 2.2. Study design

Infants underwent an echocardiographic examination and NT-proBNP test for the first time during the first 48 h of life, and echocardiography was performed weekly until the PDA closed or discharge. The optimal management method, including conservative treatment, oral ibuprofen therapy or surgical ligation, was chosen by incorporating clinical symptoms and echocardiography results. Echocardiographic indicators were recorded in detail, and clinical data were collected. Infants were screened for risk factors for PDAao and the nomogram prediction model was established.

### 2.3. Data collection

The following characteristics of enrolled patients were collected from the electronic medical record systems: (1) General patient data: gestational age at birth, birth weight, small for gestational age, sex, modes of delivery and feeding, and perinatal asphyxia; (2) Maternal data: maternal abortion times, the use of antenatal corticosteroids, premature rupture of membranes >24 h before delivery, maternal preeclampsia and gestational diabetes mellitus; (3) Echocardiographic parameters: PDA diameter, PDA maximum flow velocity, left atrium diameter, aortic diameter and LA/AO ratio, left ventricular end-diastolic diameter (LVEDD) and left ventricular end-systolic diameter (LVESD), mitral valve inflow E wave, A wave and E/A ratio, and left ventricular ejection fraction (LVEF); and (4) Clinical patient data: blood pressure on Day 1 and Day 2, pH, base excess (BE) and lactic acid levels on Day 1, plasma NT-proBNP levels in the first 48 h, platelet parameters including the platelet count, platelet distribution width, mean platelet volume, platelet-large cell ratio and thrombocytocrit level in the first 3 days, the mode of respiratory support and oxygen requirements, the application of pulmonary surfactant, the use of ibuprofen, the incidence of pneumonia, BPD, IVH, periventricular leukomalacia, feeding intolerance and NEC, and mortality before discharge.

### 2.4. Statistical analyses

SPSS software (Version 23.0, IBM, USA) and R software (Version 4.0.3) were used for statistical analysis and nomogram development. Univariate analysis was conducted for all baseline and antenatal characteristics, early vital signs and laboratory examination results, and echocardiographic variables. Continuous variables in the two groups were tested for normality using the Shapiro‒Wilk test and are presented as the mean (*x ± s*) or median (*Q1, Q3*) as appropriate. Analysis was conducted using a Student *t* test or the Mann‒Whitney *U* test. Categorical variables are expressed as percentages (%) and were compared using the *χ^2^* or Fisher exact test as appropriate. Statistical significance was defined as *p *< 0.05. Multivariate logistic regression analysis was performed for the variables that reached the significance level of *p *< 0.05 in the univariate analysis. If a significant effect was observed in the logistic model, the independent prognostic factors were determined (*p *< 0.05). Odd ratios (ORs) are used to demonstrate the correlations between factors and PDAao.

According to the final logistic regression model and by using the rms package in R version 4.0.3, the nomogram model was constructed. Internal verifications were performed for the nomogram, and the discrimination and calibration of the model were evaluated. The evaluation of the discrimination in this article was based on the index of concordance (C-index), i.e., the same number of samples was repeatedly extracted from a given database and then put back, and the internal evaluation of the resolution of the nomogram model was performed in the new generated sample. Bootstrapping with 1,000 resamples was conducted for internal evaluation. A C-index of 0.5 indicated that the model had no predictive effect. A C-index of 1 indicated that the predicted results of the model were completely concordant with the actual results. The closer the C-index was to 1, the better the predicted results of the model. Evaluation of the degree of calibration was based on the calibration plot method, which involved a comparison between the event incidence predicted by the nomogram model and the true incidence. The model was uesd to predict the risk value from low to high, segment the queue, calculate the average predicted risk value (*x*-value) in each segment and the correspondinng true risk value (*y*-axis), obtain the calibration point in each segment to draw the predicted calibration curve and the standard curve, the better the conformity of the prediction model. The nomogram matched each variable to the corresponding score, and the sum of the scores of all variables was defined as the total score. Based on regression analysis, the probability of PDAao was estimated by drawing a vertical line from the axis of the total score. Logarithmic conversion was performed for NT-proBNP levels. *p *< 0.05 were considered statistically significant, and ORs are presented with 95% confidence intervals (CIs).

### 2.5. Ethics approval

Informed consent was provided for all study participants to be enrolled in the care initiative program. This study was approved by the Medical Ethics Committee, Tongji Hospital, Tongji Medical College, Huazhong University of Science and Technology (TJ-IRB20210229).

## 3. Results

### 3.1. Baseline and antenatal characteristics

A total of 82 VLBW infants (44 males and 38 females) were included in this study, with 41 infants in the AO group and 41 infants in the NO group. The mean gestational age at birth of the AO and NO groups was 29.3 ± 1.7 weeks and 30.6 ± 2.3 weeks, respectively; the mean birth weight of the AO and NO groups was 1118.0 ± 216.4 g and 1270.7 ± 153.1 g. The gestational age at birth and birth weight were significantly lower in the AO group (*p *< 0.05). No difference was observed in the other baseline characteristics between the AO and NO groups. The baseline characteristics are listed in Table 1.

**Table 1:** Baseline and antenatal characteristics of the two groups.

Characteristics	AO group (*n* = 41)	NO group (*n* = 41)	*p* Value
Gestational age, weeks (x ± s)	29.3 ± 1.7	30.6 ± 2.3	0.007
Birth weight, g (x ± s)	1118.0 ± 216.4	1270.7 ± 153.1	0.004
Small for gestational age, *n* (%)	13 (31.7)	14 (34.1)	0.814
Males, *n* (%)	23 (56.1)	21 (51.2)	0.658
Cesarean delivery, *n* (%)	31 (75.6)	33 (80.5)	0.594
Breast feeding, *n* (%)	21 (51.2)	16 (39.0)	0.267
Perinatal asphyxia, *n* (%)	39 (95.1)	36 (87.8)	0.429
Matenal abortion times, median (Q1, Q3)	0 (0, 2.0)	1.0 (0, 2.0)	0.187
Antenatal steroids, *n* (%)	32 (78.0)	33 (80.5)	0.785
Premature rupture of membranes, *n* (%)	7 (17.1)	14 (34.1)	0.077
Maternal preeclampsia, *n* (%)	18 (43.9)	13 (31.7)	0.255
Gestational diabetes mellitus, *n* (%)	11 (26.8)	6 (14.6)	0.173
Pulmonary surfactant, *n* (%)	24 (58.5)	16 (39.0)	0.077
Ibuprofen, *n* (%)	18 (43.9)	10 (24.4)	0.062

### 3.2. Early vital signs and laboratory examinations

The systolic blood pressure and diastolic blood pressure on Day 1 and Day 2 were significantly lower in the AO group than in the NO group (*p *< 0.05). Infants in the AO group had higher plasma NT-proBNP levels in the first 48 h than those in the NO group (18,549.0 pg/ml vs. 5759.0 pg/ml, *p *< 0.001). PH, base excess (BE) and lactic acid levels on Day 1 showed no differences between the two groups (*p *> 0.05). In addition, there were no significant differences in the platelet parameters in the first 3 days between the two groups (*p *> 0.05). The factors are listed in Table 2.

**Table 2:** Early vital signs and laboratory examinations of the two groups.

Factors	AO group (*n* = 41)	NO group (*n* = 41)	*p* Value
Systolic BP D1, mmHg (x ± s)	51.1 ± 9.6	56.3 ± 8.8	0.012
Diastolic BP D1, mmHg (x ± s)	25.5 ± 7.8	29.0 ± 6.7	0.03
Systolic BP D2, mmHg (x ± s)	52.7 ± 8.3	57.2 ± 7.2	0.01
Diastolic BP D2, mmHg (x ± s)	26.6 ± 5.0	31.3 ± 5.7	<0.001
NT-proBNP, pg/ml (median (Q1, Q3))^a^	18549.0 (9793.0, 29235.0)	5759.0 (3245.5, 11156.0)	<0.001
Arterial blood gas^b^			
pH, median (Q1, Q3)	7.27 (7.22, 7.30)	7.28 (7.23, 7.31)	0.802
-BE, mmol/L (median (Q1, Q3))	5.9 (4.2, 8.5)	5.3 (4.2, 7.1)	0.25
Lac, mmol/L (median (Q1, Q3))	3.5 (2.1, 4.3)	2.7 (1.9, 3.8)	0.266
Platelet parameters^c^			
PLT, 10^9^/L (x ± s)	198.0 ± 67.5	213.6 ± 66.8	0.294
PDW, fL (median (Q1, Q3))	11.8 (10.5, 12.7)	11.1 (10.2, 12.2)	0.312
MPV, fL (x ± s)	10.6 ± 0.8	10.3 ± 0.8	0.164
Platelet-large cell ratio, % (x ± s)	28.3 ± 5.5	26.9 ± 6.5	0.28
PCT, % (x ± s)	0.21 ± 0.06	0.22 ± 0.07	0.384

BP, blood pressure. D1, Day 1. D2, Day 2. Lac, lactic acid. PLT, platelet. PDW, platelet distribution width. MPV, mean platelet volume. PCT, thrombocytocrit.

^a^ During the first 48 h.

^b^ During the first day.

^c^ During the first 3 days.

### 3.3. Echocardiographic parameters in the first 48 h of life

For infants in the AO group, the PDA diameter (2.3 mm vs. 2.0 mm) and LA/AO ratio (1.60 vs. 1.46) were larger, and the PDA maximum flow velocity (1.3 m/s vs. 1.9 m/s) was lower than that in the NO group (*p *< 0.05). The differences in the other echocardiographic parameters during the first 48 h of life were not significant (*p *> 0.05, Table 3).

**Table 3:** Echocardiographic parameters of the two groups in the first 48 h.

Parameters	AO group (*n* = 41)	NO group (*n* = 41)	*p* Value
PDA diameter, mm (median (Q1, Q3))	2.3 (2.0, 3.0)	2.0 (1.9, 2.4)	0.01
PDA maximum flow velocity, m/s (x ± s)	1.3 ± 0.5	1.9 ± 0.5	<0.001
LA diameter, mm (x ± s)	7.8 ± 1.4	7.5 ± 1.0	0.256
AO diameter, mm (x ± s)	4.9 ± 0.7	5.2 ± 0.7	0.101
LA/AO ratio, x ± s	1.60 ± 0.21	1.46 ± 0.15	0.001
LVEDD, cm (median (Q1, Q3))	12.5 (10.6, 13.3)	12.8 (11.8, 13.9)	0.106
LVESD, cm (median (Q1, Q3))	8.6 (7.1, 9.1)	8.8 (8.2, 9.5)	0.215
E wave, m/s (median (Q1, Q3))	0.39 (0.34, 0.49)	0.42 (0.36, 0.51)	0.257
A wave, m/s (median (Q1, Q3))	0.45 (0.40, 0.56)	0.47 (0.38, 0.57)	0.663
E/A ratio, median (Q1, Q3)	0.87 (0.80, 0.97)	0.92 (0.82, 1.09)	0.246
LVEF, median (Q1, Q3)	65.0 (63.0, 66.0)	63.0 (62.0, 66.0)	0.114

LA, left atrium; AO, aortic; LVEDD, left ventricular end-diastolic diameter; LVESD, left ventricular end-systolic diameter; LVEF, left ventricular ejection fraction.

### 3.4. Multivariable logistic regression analysis of PDAao

Ten variables showed significant differences in the univariate analysis and were further included in the multivariable logistic regression analysis. As shown in Table 4, 4 variables were considered as independent predictors, including the PDA diameter (OR = 1.194, 95% CI 1.009–1.414, *p *= 0.039), PDA maximum flow velocity (OR = 0.077, 95% CI 0.009–0.670, *p *= 0.02), LA/AO ratio (OR = 1.925, 95% CI 1.118–3.315, *p *= 0.018) and NT-proBNP level (OR = 2.535, 95% CI 1.297–4.953, *p *= 0.007).

**Table 4:** Multivariable logistic regression analysis of PDAao.

Variables	*β*	*OR*	95% CI	*p* Value
Gestational age	−0.108	0.898	0.555–1.452	0.661
Birth weight	0.001	1.001	0.995–1.007	0.722
(PDA diameter) × 10^a^	0.178	1.194	1.009–1.414	0.039
PDA maximum flow velocity	−2.565	0.077	0.009–0.670	0.020
(LA/AO ratio) × 10^a^	0.655	1.925	1.118–3.315	0.018
Log_2_ (NT-proBNP)^a^	0.930	2.535	1.297–4.953	0.007
Systolic BP, D1	0.056	1.058	0.913–1.225	0.454
Diastolic BP, D1	−0.013	0.987	0.802–1.216	0.906
Systolic BP, D2	0.093	1.097	0.865–1.392	0.445
Diastolic BP, D2	−0.261	0.771	0.574–1.035	0.084

^a^ The PDA diameter, LA/AO ratio and NT-proBNP were, respectively converted, in which the PDA diameter and LA/AO ratio were increased by 10 times, and logarithmic conversion was performed for NT-proBNP.

### 3.5. Nomogram model

**Figure 1:**
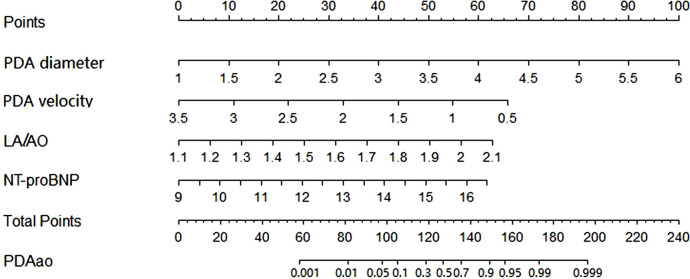
Nomogram model of PDAao the probability of PDAao was calculated by drawing a line to the points on the top axis for each of the variables. The points for four variables were added and located on the total points line. Then a vertical line was drawm from the total points line to the PDAao line to obtain the probability of PDAao. NT-proBNP was performed logarithmic conversion.

**Figure 2:**
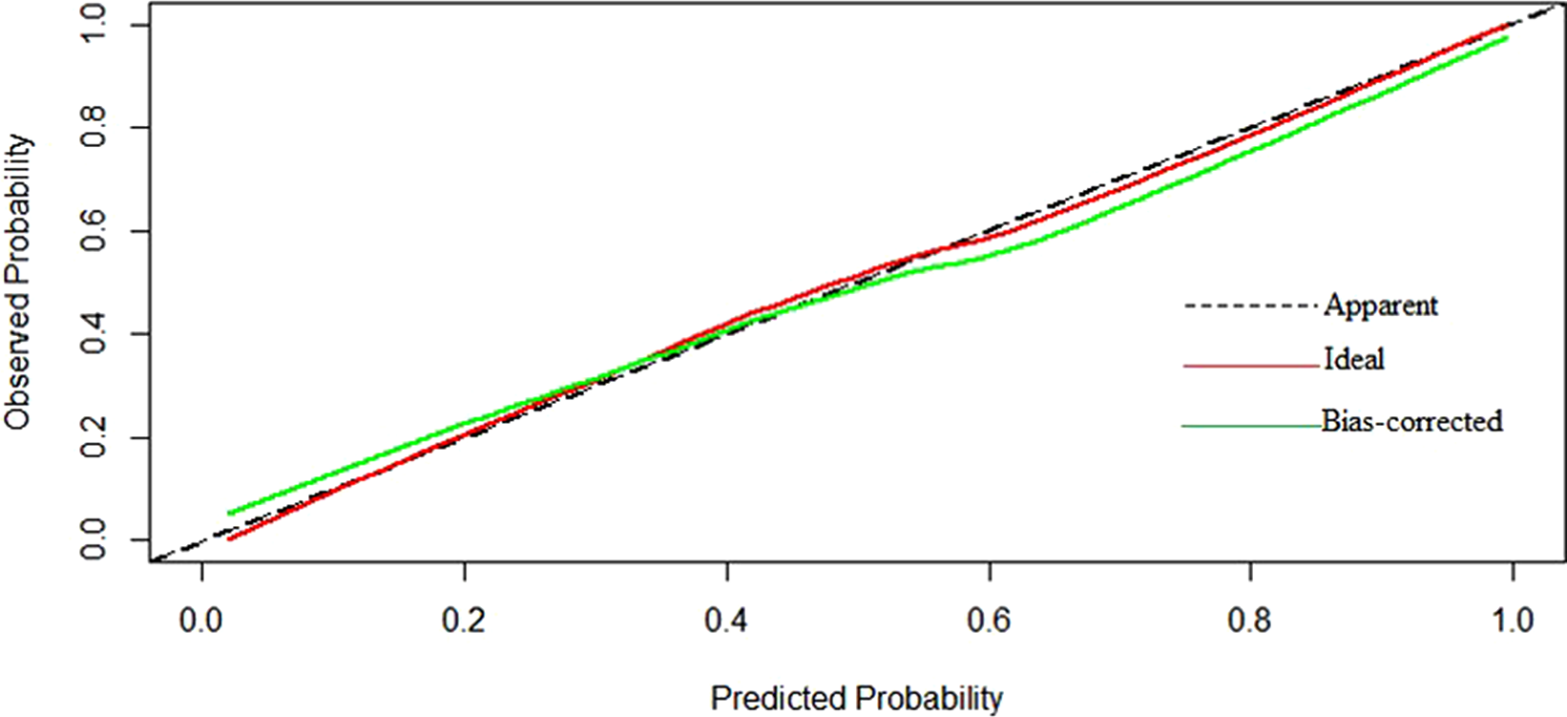
Calibration curves of PDAao nomogram.

Based on multivariable logistic regression results, the PDA diameter, PDA maximum flow velocity, LA/AO ratio and NT-proBNP level were incorporated into the nomogram model constructed to predict the risk of PDAao. The prediction nomogram model for PDAao is shown in Figure 1. The C-index value of the nomogram model was 0.917 (95% CI 0.859–0.975), and the corrected C-index value was 0.894, which indicated a good discrimination capacity and predictive value. The calibration curves in Figure 2 showed highly consistency and indicated good correspondence between the event incidence predicted by the nomogram model and the true incidence of PDAao.

## 4. Discussion

In this study, severe complications were closely related to PDA and greatly affected the survival rate and quality of life of VLBW infants, including severe BPD, stage IV with IVH, NEC Bell stage II and above and death. These complications were selected as the study endpoints to explore whether early sensitive indicators of PDA could predict the occurrence of adverse outcomes and improve the clinical outcomes of VLBW infants.

We concluded that the PDA diameter, PDA maximum flow velocity, LA/AO ratio and NT-proBNP level during the first 48 h are independent risk factors for the occurrence of PDAao in VLBW infants. The PDA diameter and PDA maximum flow velocity can reflect the shunt volume of ductus arteriosus. When the PDA diameter grows larger or the maximum velocity across the PDA lowers (indicating a lack of ductal constriction and an unrestricted flow pattern in the absence of significant pulmonary hypertension), the shunt volume of the PDA and pulmonary blood flow increases (24). In addition, a large left-to-right shunt of ductus arteriosus can lead to increased volume overload in the left atrium (AO), which gradually dilates, and if this process persists, it leads to dilatation of the left ventricle due to the increased preload, especially in the absence of a large intra-atrial shunt (25). The aortic root diameter (AO) is a relatively fixed structure and does not dilate due to the left-to-right shunt. Hence, the LA/AO ratio is only related to the ductus arteriosus diameter and shunt volume and is less affected by age (25, 26).

With the establishment of respiration after birth, the pulmonary vascular pressure decreases rapidly, while the volume load and pressure load of the left heart increase rapidly, and the level of NT-proBNP increases (27). If PDA exists, the left ventricular volume load further increases, and the level of NT-proBNP also significantly increases (21).

Studies have shown that a larger PDA shunt and higher LA/AO ratio are associated with higher NT-proBNP levels (18). Some studies have suggested that a decrease of the NT-proBNP level ≥80% on the third day after birth can sensitively predict PDA closure in extremely low birth weight infants (28). In this study, the results showed that NT-proBNP levels change significantly within 48 h after birth, and an increasing NT-proBNP level is an independent risk factor for PDAao.

Several studies have attempted to predict the incidence of severe complications in infants with PDA. Sehgal A et al. (29) included 52 preterm infants (<32 weeks) who were diagnosed with PDA and treated with ibuprofen (average treatment time was 7 days after birth), and the selected preterm infants were divided into a BPD group and non-BPD group. The PDA combined score was established according to echocardiographic parameters within 48 h before treatment, including the PDA shunt diameter, PDA maximum shunt velocity, PDA/left pulmonary artery diameter, LA/AO ratio, and mitral valve E/A ratio. The study showed that the PDA combined score in the BPD group was significantly higher than that in the non-BPD group (*P* < 0.05). In this paper, echocardiographic parameters within 48 h before treatment were selected to explore the relationship between the occurrence of BPD and the echocardiographic indicators of PDA. The timing of echocardiography and endpoint events were different from those in our study. Schena F et al. (6) included 242 preterm infants with a gestational age <28 weeks and divided them into BPD/death group (*n* = 105) and control group (*n* = 137). The echocardiographic results of the enrolled infants from birth to 36 weeks of corrected gestational age were reviewed, and the association between PDA and late BPD/death events was evaluated according to the “PDA staging system” proposed by McNamara PJ and Sehgal A (30). The “PDA staging system” mainly classifies PDA into none, mild, moderate, and severe according to the diameter and direction of the PDA shunt, LA/AO ratio, and E/A ratio. The duration of moderate to severe PDA in the BPD/death group was significantly longer than that in the control group (4.8 days vs. 2.3 days, *P* < 0.001). This article only discussed the relationship between BPD/death and PDA, and classified the severity of PDA according to echocardiographic parameters.

Compared to the above studies, in this study, we selected preterm infants with a birth weight less than 1500 g, recorded the clinical data in detail, including baseline and antenatal characteristics, and applied PS and treatment for hsPDA to clarify the influence of the above factors on adverse outcomes of PDA. Then, we selected sensitive echocardiographic parameters within 48 h after birth to emphasize the predictive ability of early PDA-related sensitive indicators for adverse outcomes. We not only selected the PDA shunt diameter but also included the PDA maximum shunt velocity and LA/AO ratio to comprehensively evaluate the hemodynamic changes in infants with PDA. In addition, the blood NT-proBNP level, which is closely related to PDA, was added to reduce the possibility of human error caused by ultrasound operation to more accurately predict the occurrence of PDAao. And the endpoints of our study included severe BPD, grade IV with IVH, NEC Bell stage II or above, and death, which were greatly affected by the presence of PDA.

We developed a nomogram prediction model for PDAao in VLBW infants; and internal verifications were performed for the nomogram, and the discrimination and calibration of the model were evaluated by C-index and calibration curve. The results of this study suggest that the PDAao nomogram model had high discrimination and good prediction accuracy, and the event incidence predicted by the nomogram model and the true incidence of PDAao were in good agreement. According to the nomogram model, a very low birth weight infant with a 50% risk of PDAao has a corresponding total score of 127. When a child's PDA diameter is greater than 2.5 mm, the PDA maximum shunt velocity is less than 2 m/s, the LA/AO ratio is greater than 1.6, and the NT-proBNP level is greater than 8000 pg/ml, the corresponding scores for each single item are 30, 32, 31 and 32, respectively. The total score is close to 127, and the risk of PDAao is increased. However, the variables in this model are all continuous numerical variables, which could be considered to be transformed into categorical variables in later stages, which is more conductive to the clinical application of the nomogram model.

## 5. Conclusion

This study suggests that sensitive PDA echocardiographic parameters within 48 h after birth, including the PDA shunt diameter, PDA maximum shunt velocity, LA/AO ratio, and blood NT-proBNP level, can be used to predict PDAao in VLBW infants. It has a good prediction value for the adverse outcomes of PDA in VLBW infants (moderate to severe BPD, grade IV with IVH, NEC Bell stage II or above, and death).

## Data Availability

The original contributions presented in the study are included in the article/Supplementary Material, further inquiries can be directed to the corresponding author/s.
